# Case Report: Early surgical intervention for epilepsy with mild MOGHE: timing and clinical efficacy

**DOI:** 10.3389/fped.2026.1790096

**Published:** 2026-04-21

**Authors:** Yaning Sun, Liuyin Chen, Mei Jin, Fan Yang, Yakun Du, Jiangshun Fang, Baoguang Li, Zhixuan Sun, Lingyan Wang, Zhenghai Cheng, Zhiguo Yang, Yi Qu

**Affiliations:** 1 Department of Neurosurgery, Hebei Province of Children’s Hospital, Shijiazhuang, Hebei, China; 2 The Fifth People’s Hospital of Hebei Province, Shijiazhuang, Hebei, China; 3 Hebei Children’s Health and Disease Clinical Medical Research Center, Shijiazhuang, Hebei, China

**Keywords:** early surgical intervention, epilepsy, epileptic spasms, MOGHE, surgical timing

## Abstract

**Background:**

Mild malformation of cortical development with oligodendroglial hyperplasia in epilepsy (MOGHE) is a new histopathological entity identified in resected brain tissue from patients with drug-resistant epilepsy. Clinically, this epilepsy subtype most commonly presents as epileptic spasms in early childhood. Conventional anatomical-electro-clinical approaches struggle to accurately delineate the epileptogenic zone, posing substantial challenges to clinical management.

**Case description:**

This paper reports a 2-year-and-11-month-old male infant who developed significant intellectual and language regression merely 3 months after the onset of epileptic seizures. Scalp video-electroencephalography (VEEG) demonstrated generalized epileptiform discharges, cranial magnetic resonance imaging (MRI) revealed abnormal signals in the left frontal lobe, and fused cranial 18F-fluorodeoxyglucose positron emission tomography (PET)-MRI imaging showed no obvious hypometabolic foci. Following oral vigabatrin administration, clinical seizures were absent, yet neurodevelopmental regression progressed continuously. For children with early-onset developmental regression, a clinical decision-making dilemma exists: whether to continue medication adjustment and await fulfillment of traditional drug-resistant epilepsy criteria before surgery, or to implement active early surgical intervention.

**Conclusion:**

Through full-course follow-up of this child with MOGHE-associated epilepsy, this study integrated the evolution of clinical symptoms, electroencephalographic characteristics, imaging findings, and treatment responses to explore the rationale for surgical timing selection. The results suggest that for childhood epilepsy caused by definite cerebral structural abnormalities (particularly MOGHE) that has already led to obvious neurodevelopmental regression, active early surgical treatment should be considered to prevent disease progression and create favorable conditions for subsequent rehabilitation.

## Introduction

Epilepsy is one of the most common neurological diseases in childhood, with a particularly high incidence in early infancy (1). A recent systematic review and meta-analysis by Sultana et al. (2) reported that the cumulative incidence of drug-resistant epilepsy in children is as high as 25.0% (95% CI: 16.8–34.3), significantly exceeding the 14.6% observed in adult or mixed-age cohorts. Long-term epileptic seizures not only severely impact children's quality of life but also cause developmental delay or regression of cognitive, language, and motor functions. This adverse effect is particularly pronounced when seizures onset in infancy, a critical period for brain development. Makridis et al. (3) reported that 84.6% of infants who underwent epilepsy surgery within 6 months of birth had pre-existing cognitive impairment (developmental quotient <85), indicating early-onset seizures cause significant developmental damage. Subsequent multicenter studies confirmed that all seizure-free infants post-surgery exhibited improved development despite surgical risks (4), and a recent meta-analysis by Hendrawan et al. (5) emphasized that postoperative seizure freedom is crucial for infant developmental outcomes.

Clinically, some children develop drug-resistant epilepsy despite standardized pharmacotherapy, at which point surgery becomes an important intervention. However, surgical timing selection remains a clinical dilemma, especially in early infancy, requiring a comprehensive balance between epilepsy-induced long-term brain developmental damage and surgical risks (6).

In recent years, mild malformation of cortical development with oligodendroglial hyperplasia in epilepsy (MOGHE) has been gradually recognized as a novel histopathological entity (7, 8), and officially classified as a special subtype of focal cortical dysplasia (FCD) by the International League Against Epilepsy (ILAE) in 2022 (9). MOGHE predominantly presents as infantile-onset epileptic spasms, with VEEG typically showing generalized or multifocal discharges. Conventional cranial MRI fails to clearly identify structural abnormalities, and even fused PET-MRI often shows no significant hypometabolism, making epileptogenic zone localization difficult and posing great challenges to diagnosis and treatment (10, 11).

The international diagnostic standard for drug-resistant epilepsy typically requires frequent seizures despite standardized treatment with two antiepileptic drugs for more than one year, after which preoperative evaluation and surgery are considered (12). However, MOGHE-associated epilepsy causes significant early neurodevelopmental regression with limited pharmacotherapeutic efficacy. Waiting for traditional drug-resistant epilepsy criteria to be met may result in missed optimal treatment windows and irreversible neurological damage (11). Thus, the feasibility and approach of early surgical intervention for this occult structural epilepsy subtype have become a key unresolved issue in childhood epilepsy management.

By analyzing a single case of MOGHE-associated epilepsy treated with early surgery (prior to meeting traditional drug-resistant epilepsy criteria), this study explores the feasibility and clinical significance of early surgical decision-making based on neuroimaging and electrophysiological characteristics, aiming to provide new clinical insights for treatment timing selection in such children. Early identification and intervention may be the key to preventing disease progression and improving long-term prognosis.

## Case description

A 2-year-and-8-month-old male infant who was previously well experienced his first unprovoked epileptic seizure during the awake period. Seizure manifestations included slight backward body leaning and bilateral upper limb elevation, each lasting 1–2 s, occurring in clusters of 3–4 episodes, with 1–2 clusters daily. Interictal mental status was normal, without accompanying fever, vomiting, diarrhea, or rash. The family initially did not seek medical attention; five days later, seizure frequency increased to 3–4 clusters daily with the same semiology, prompting hospital admission.

**Figure 1:**
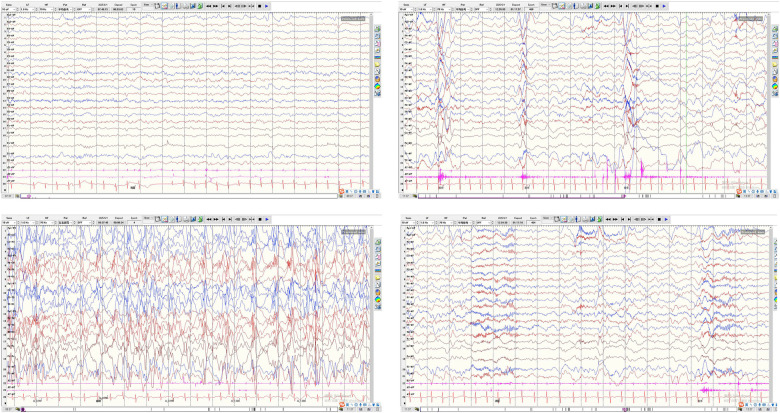
Video-electroencephalography (VEEG) findings at initial presentation without antiepileptic drug administration in a child with MOGHE-associated epilepsy.

Physical examination at initial presentation showed clear consciousness, normal mental response, and language delay (able to utter no more than 4 words). Skull shape and head circumference were normal; bilateral pupils were equal, round, and reactive to light. No obvious positive signs were found in the cardiopulmonary, abdominal, or nervous systems. The muscle strength and tone of the limbs were normal. VEEG showed generalized spike waves with hypsarrhythmia (Figure 1). Initial treatment with oral sodium valproate (28 mg/kg.d for a 14 kg infant) failed to achieve adequate seizure control.

**Figure 2:**
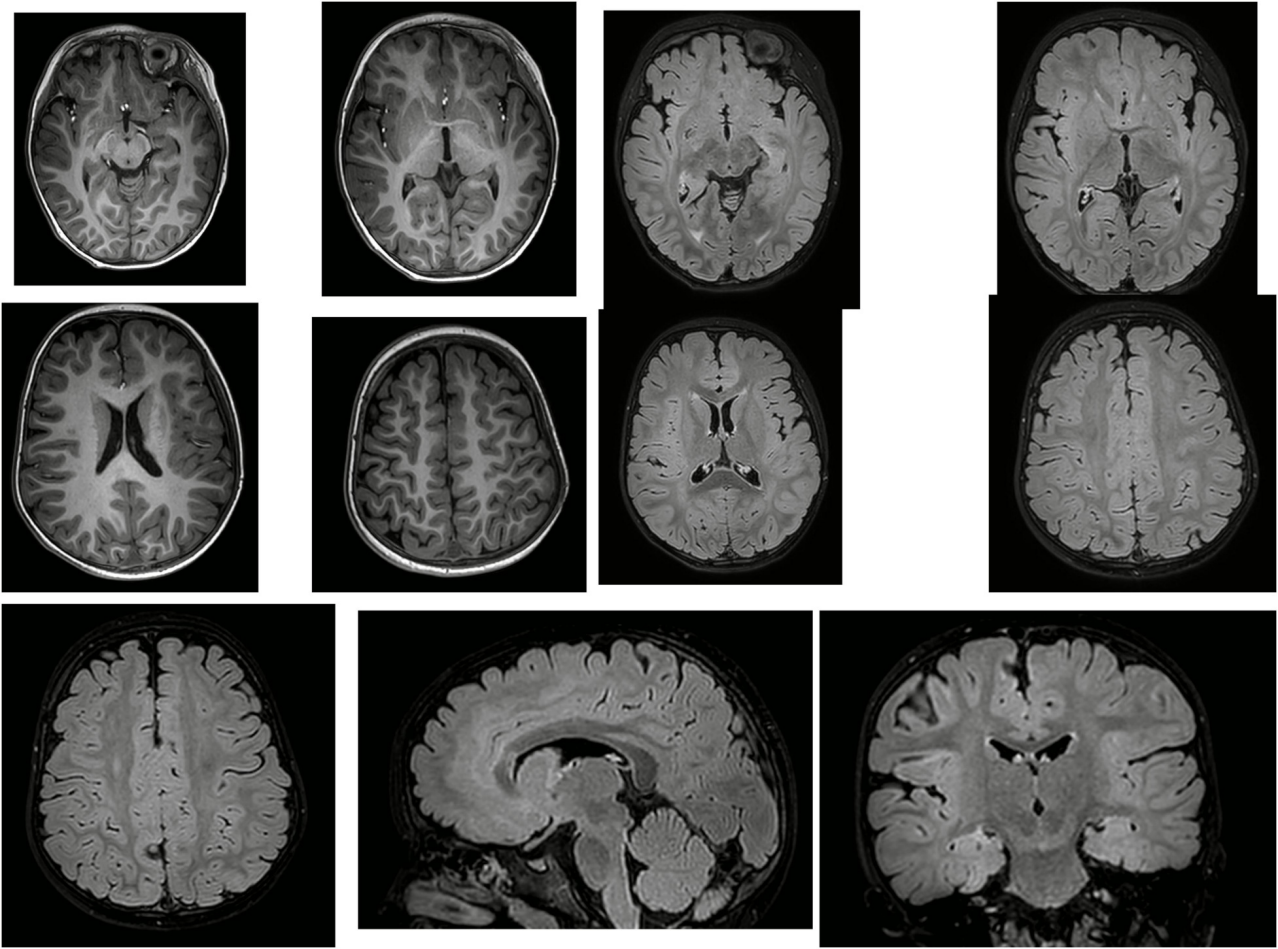
Cranial MRI T1-weighted imaging (T1WI): blurred gray-white matter junction in the left frontal lobe. Cranial MRI (T2FLAIR): irregular hyperintense signals in the left frontal lobe (predominantly medial aspect). Cranial MRI T2FLAIR (sagittal/axial/coronal): diffuse hyperintense signals in the entire left frontal lobe.

**Figure 3:**
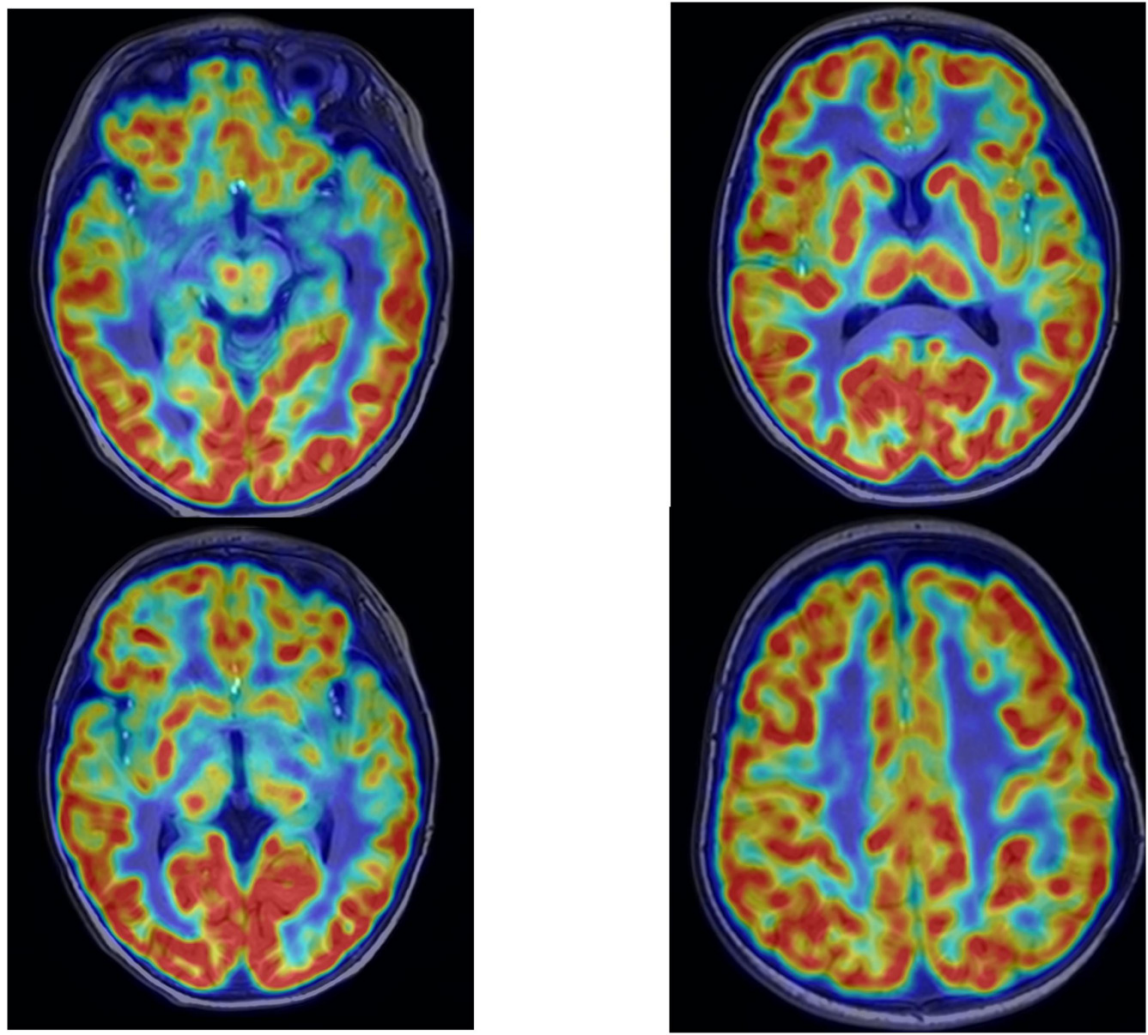
Cranial 18F-FDG PET-MRI fusion: mild hypometabolism in the medial left frontal lobe without significant epileptogenic hypometabolic foci.

Approximately one week later, vigabatrin (53 mg/kg.d for a 14 kg infant) was added at follow-up. The parents reported resolution of clinical seizures, and cranial MRI was performed concurrently, with the radiology department reporting highly suspected mild abnormal signals in the left frontal lobe (Figure 2). Following this imaging finding, the family consented to further evaluation, including 18F-fluorodeoxyglucose PET fused with cranial MRI. Fused images showed no significant hypometabolic area, only mild hypometabolism in the medial left frontal lobe (Figure 3).

Genetic testing revealed a possibly relevant but unproven pathogenic variant: FGF13 (chrX:137793098), c.68A>G (p.Asn23Ser), associated with (1) developmental and epileptic encephalopathy type 90 (XLD, XLR); (2) X-linked intellectual developmental disorder type 110 (XLR).

In view of the left frontal lobe abnormal signals on MRI, absent definite epileptogenic zone hypometabolism on PET-MRI fusion, and parental report of resolved clinical seizures after vigabatrin addition, the family expressed concerns regarding surgical risks and uncertain postoperative seizure control, and thus chose to continue pharmacotherapy and clinical observation.

However, 3 months after treatment (at 2 years and 11 months of age), despite the absence of clinical seizures, the child developed significant intellectual and language regression, only able to utter single syllables such as “baba” and “mama”. The child's developmental quotient was assessed using the Gesell Developmental Schedules, a classic tool for evaluating infant development that excels in detecting and quantifying subtle early neurodevelopmental abnormalities via a multidimensional, refined assessment system.

The scale divides development into five independent domains—adaptability, gross motor, fine motor, language, and personal-social—and calculates a developmental quotient for each, generating an individualized developmental profile that precisely identifies impaired domains. This is particularly important for children with epilepsy, whose developmental regression often presents with interdomain heterogeneity. The schedules are age-stratified and highly sensitive for infants under three years, enabling detection of “hidden” developmental stagnation even during pharmacotherapy. Moreover, the developmental quotient, as a quantitative measure, serves as a preoperative baseline and provides objective evidence for postoperative therapeutic efficacy evaluation, strongly supporting clinical decision-making regarding early surgical intervention and its potential impact on neurodevelopmental outcomes.

Assessment results indicated moderate developmental delay in adaptive behavior and language abilities, and mild developmental delay in gross motor and personal-social domains (Table 1). The Epilepsy Center of our hospital organized a multidisciplinary discussion (including Neurology, Neurosurgery, Neuroelectrophysiology, Imaging, Psychology and Behavior, Pathology, and Rehabilitation). Based on the child's epileptic spasms, left frontal lobe discharge onset on VEEG, abnormal MRI signals, and progressive neurodevelopmental regression, left frontal lobe structural lesions with highly suspected MOGHE-associated epilepsy were diagnosed.

**Table 1:** Gesell developmental schedules assessment results of the child with MOGHE-associated epilepsy at 3 months after epilepsy onset (2 years and 11 months old).

Developmental Domain	Developmental Age (DA, months)	Developmental Quotient (DQ)	Evaluation
Adaptability	15.40	45	Moderate Developmental Delay
Gross Motor	21.00	62	Mild Developmental Delay
Fine Motor	17.03	50	Moderate Developmental Delay
Language	18.20	54	Moderate Developmental Delay
Personal-Social	19.37	57	Mild Developmental Delay
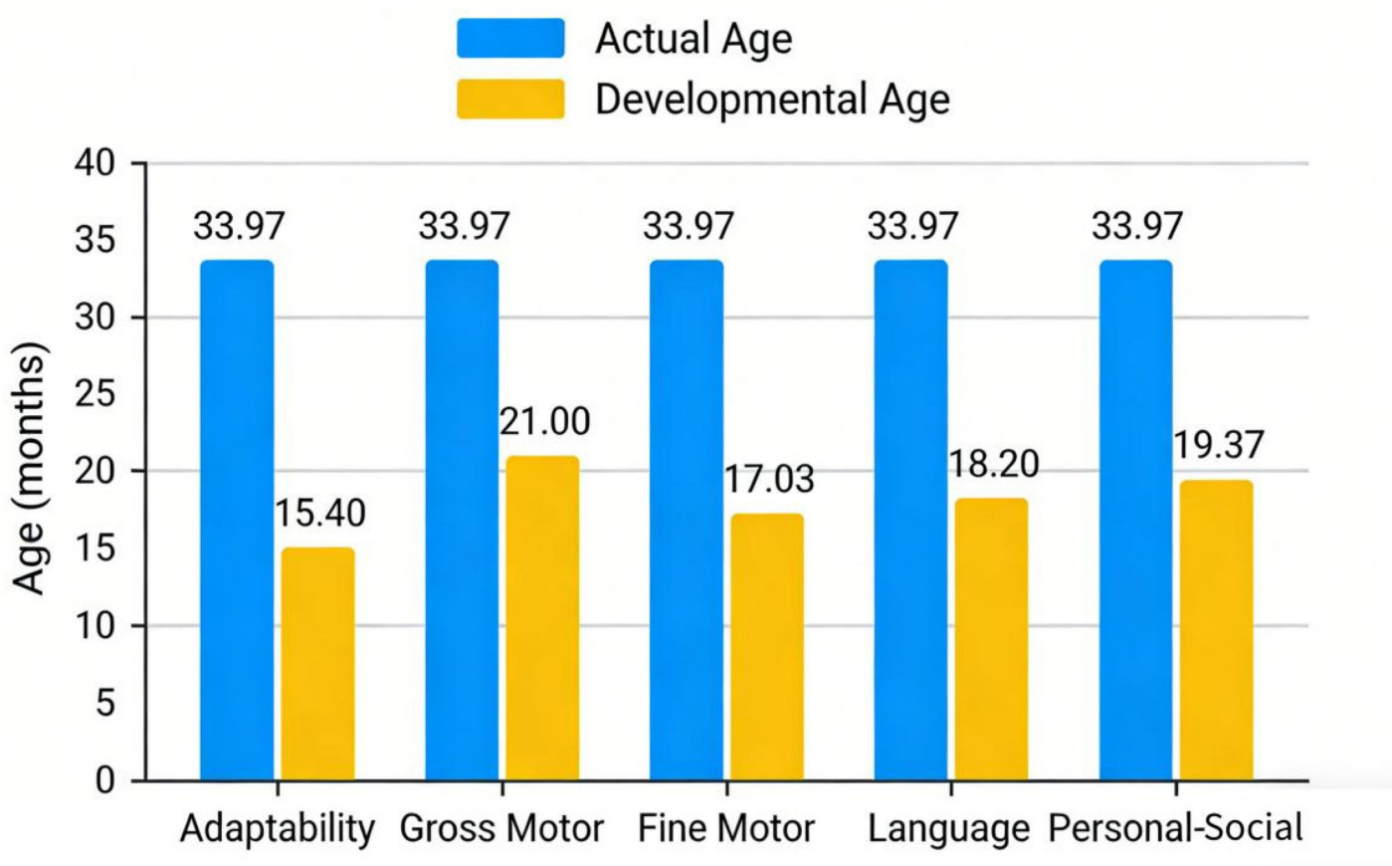			
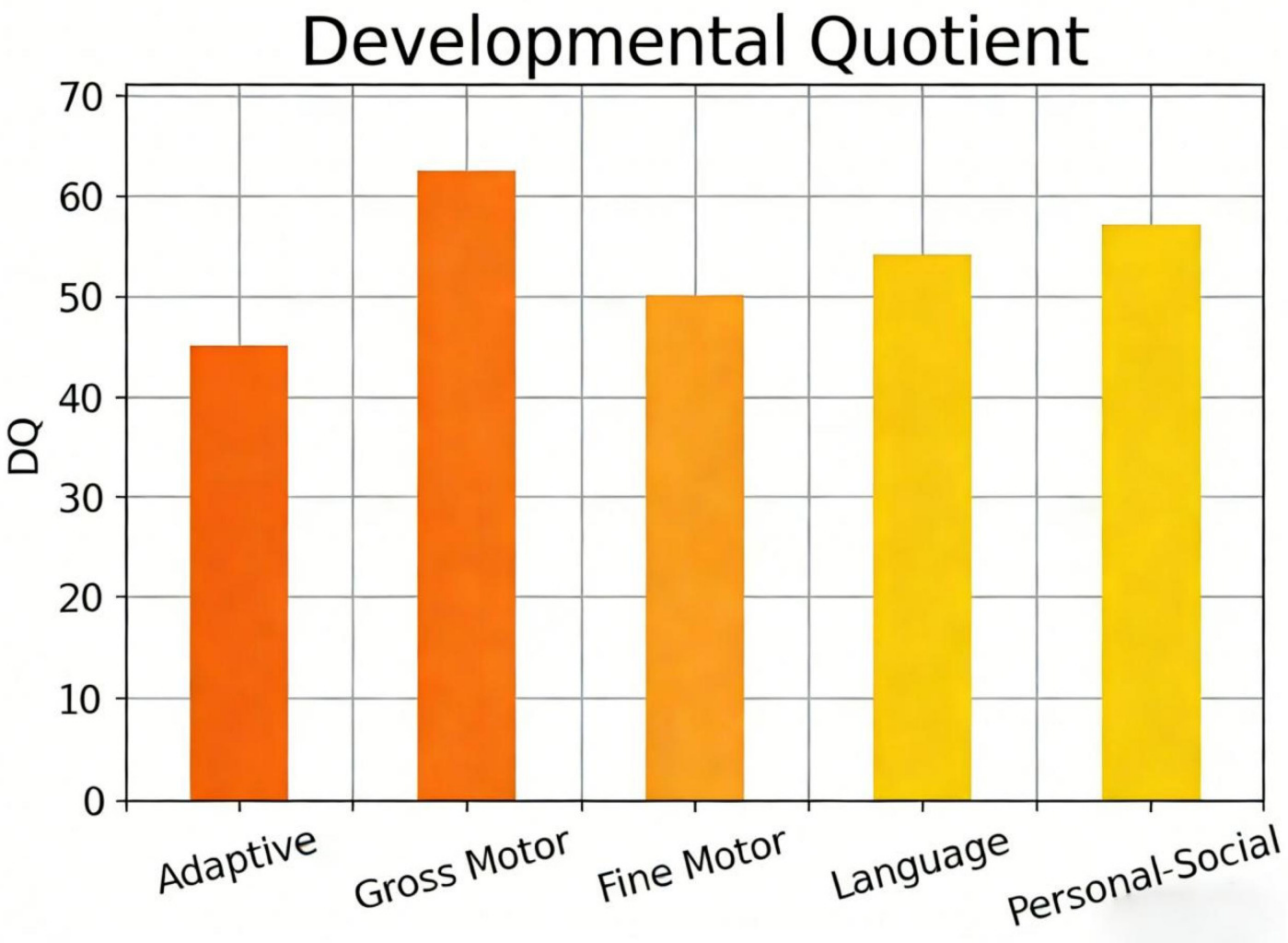			

**Figure 4:**
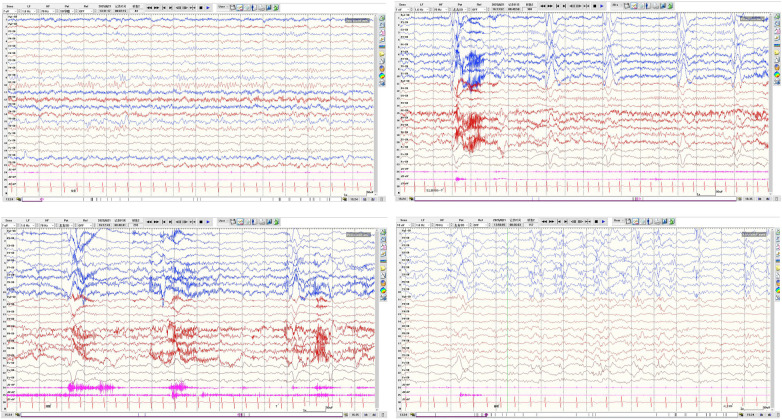
Preoperative follow-up VEEG (3 months after dual antiepileptic drug treatment): left-dominant asymmetric hypsarrhythmia and ictal epileptic spasms.

Although no overt seizures were observed during pharmacotherapy, continuous neurodevelopmental regression suggested subclinical or subtle seizure activity. Preoperative repeat VEEG recorded 2 brief epileptic spasm episodes (mild movements, indistinguishable to the naked eye) within 5 h of monitoring, with hypsarrhythmia predominantly in the left hemisphere (Figure 4), further supporting a left frontal lobe epileptogenic zone. After full informed consent and communication regarding the condition and surgical necessity, left frontal lobectomy was planned and performed.

## Operation procedures

The surgery was performed via a left frontotemporoparietal U-shaped incision for craniotomy. Intraoperatively, disorganized frontal cortical structure was observed, and electrocorticography (ECoG) indicated burst suppression in the left frontal lobe. In accordance with the preoperative plan, and with strict protection of the central region and supplementary motor area, the following procedures were completed step-by-step: resection of the posterior part of the inferior frontal gyrus, frontal base disconnection, ventricular opening, anterior corpus callosum disconnection, and partial aspiration of the anterior insula, followed by basal ganglia disconnection.

**Figure 5:**
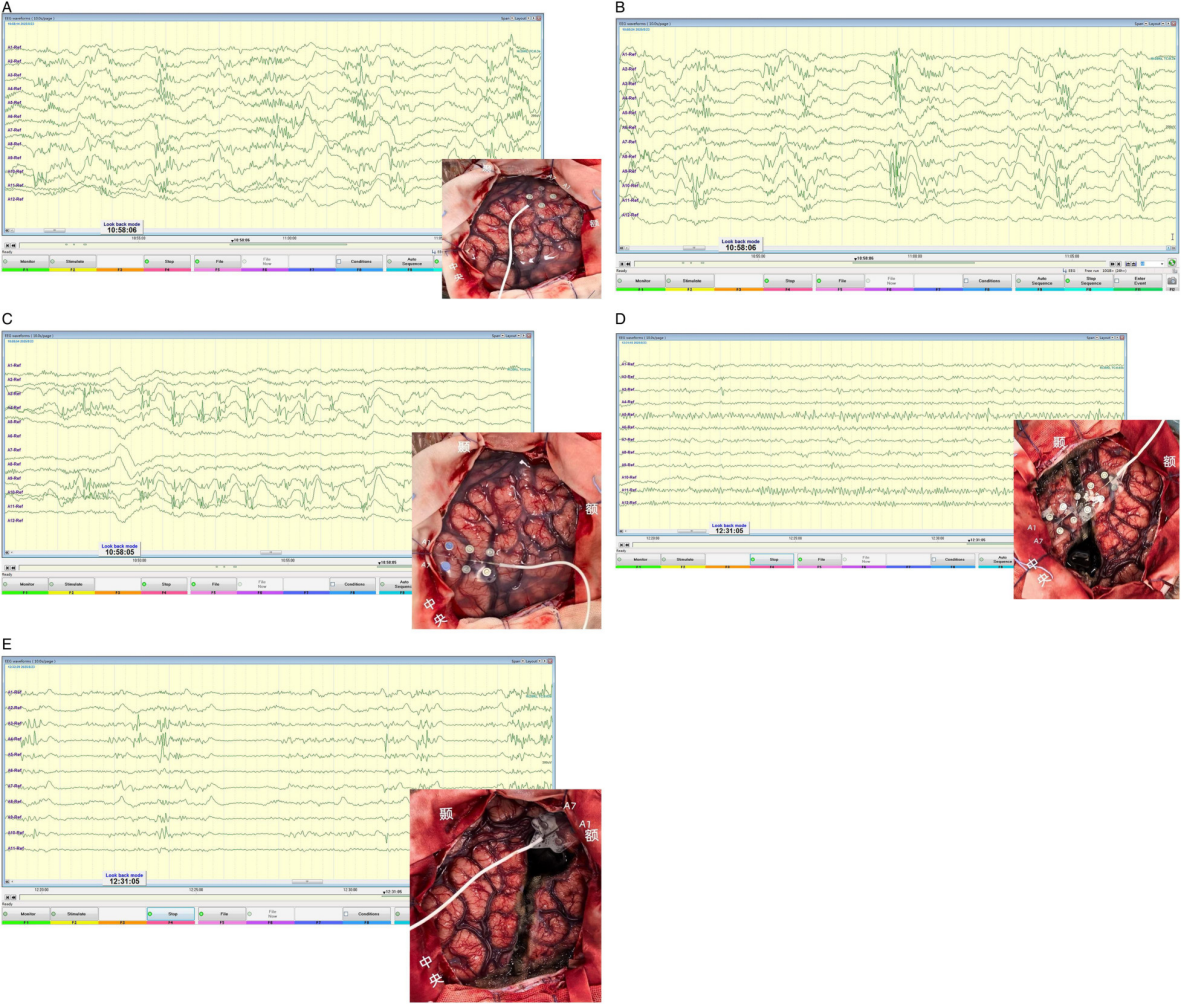
Intraoperative electrocorticography (ECoG): burst suppression in the left frontal lobe and disappearance of abnormal discharges after disconnection surgery.

**Figure 6:**
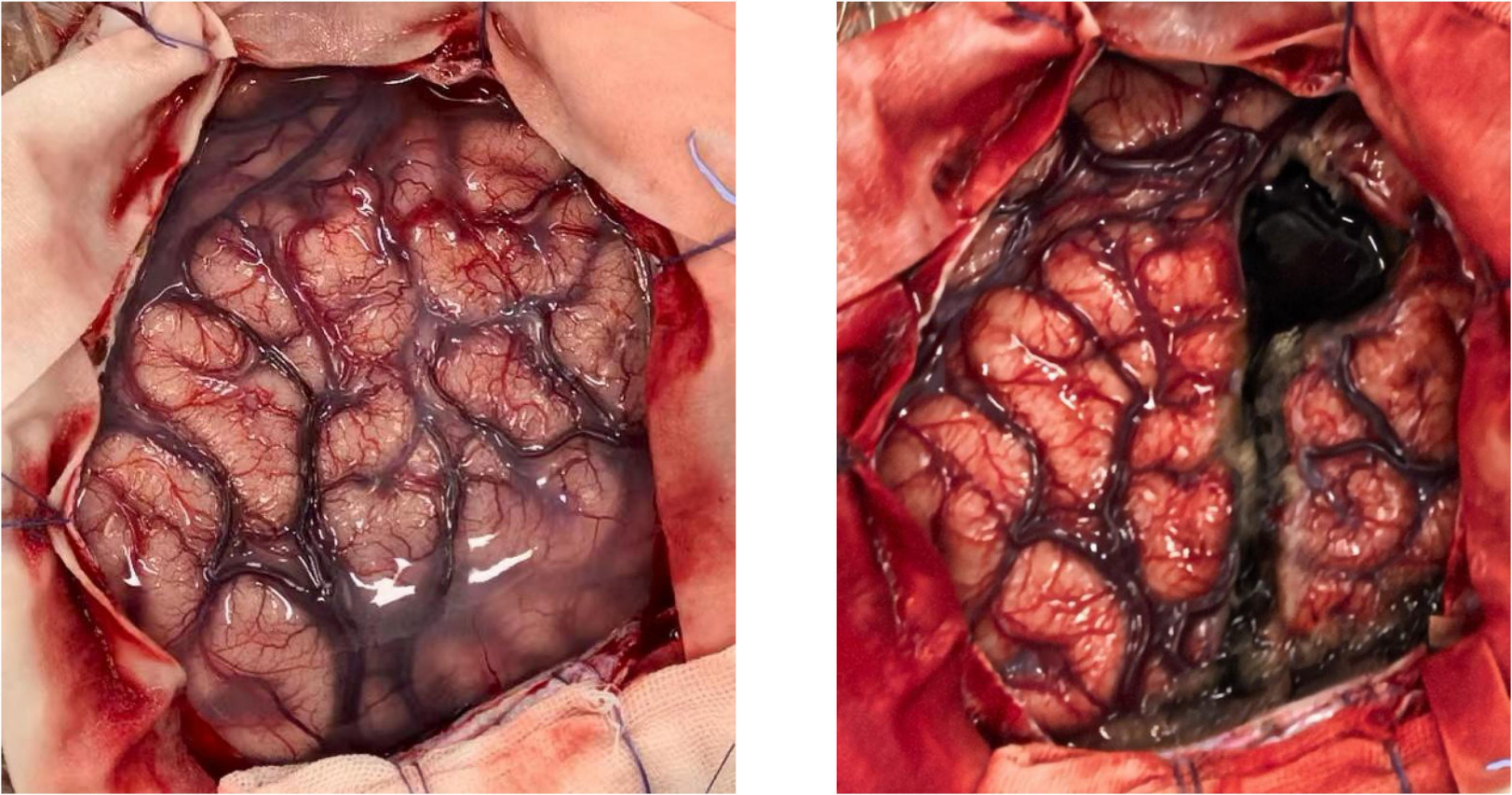
Surgical procedure for left frontal lobe disconnection: preoperative lesion location and postoperative complete disconnection of the prefrontal lobe (with central region protection).

Important neurovascular structures including the olfactory nerve were protected intraoperatively. Post-disconnection ECoG confirmed the disappearance of original abnormal discharges. The surgical field was thoroughly hemostatic; instruments were counted and verified, and the incision was sutured layer by layer. The surgery was uneventful (Figures 5, 6).

**Figure 7:**
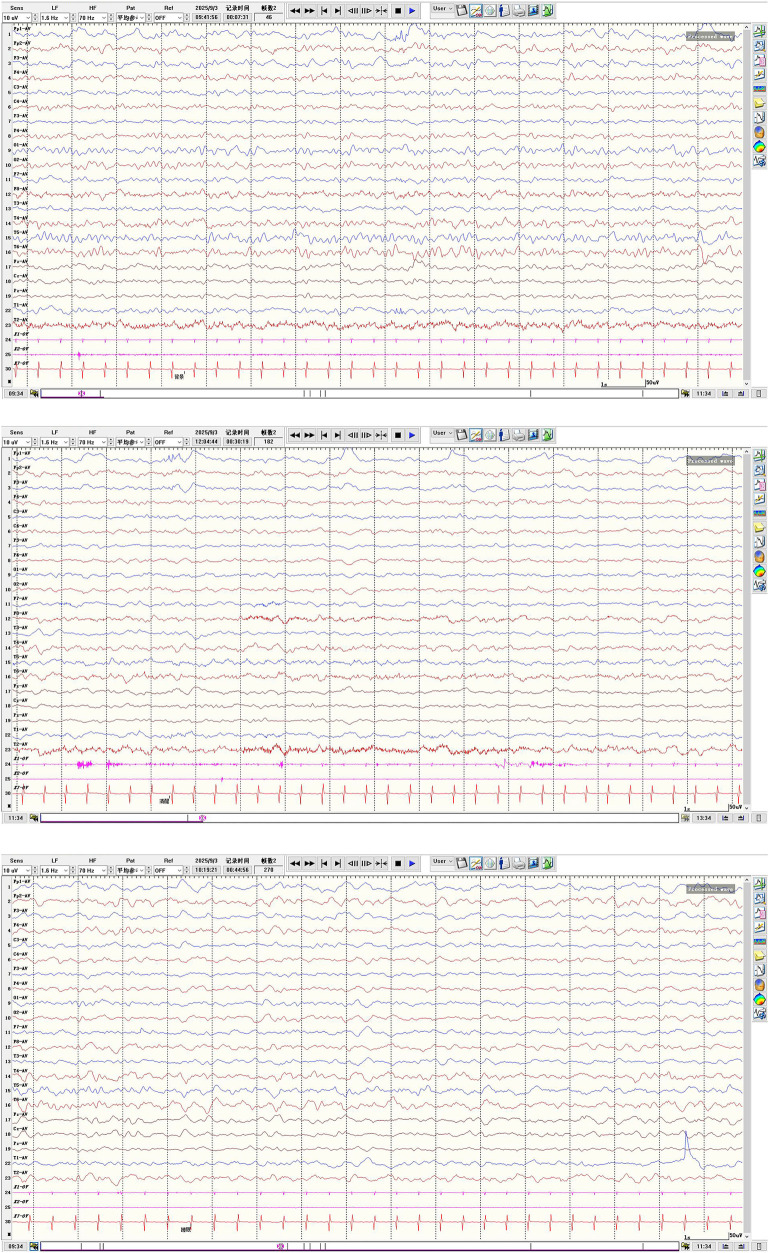
Postoperative VEEG (11 days after left frontal lobe disconnection): sparse scattered abnormal discharges in the left frontal region.

**Figure 8:**
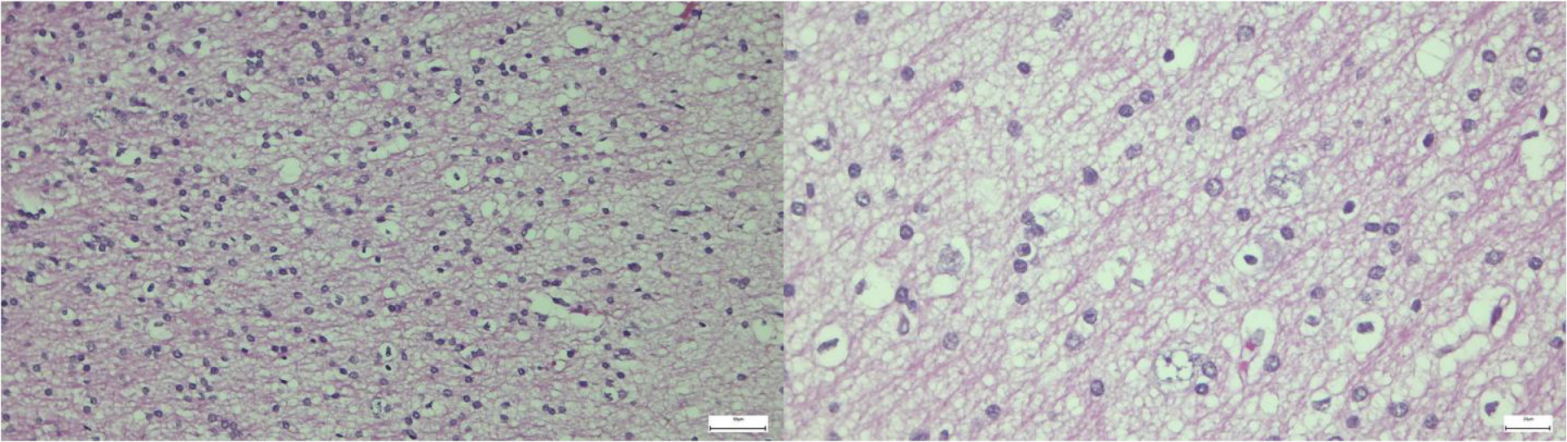
Pathological findings of resected brain tissue: clustered oligodendroglial hyperplasia confirming the diagnosis of MOGHE.

Postoperatively, the child had an uneventful recovery, and cranial CT reexamination showed stable intracranial conditions. Postoperative VEEG showed near-complete resolution of abnormal discharges (Figure 7). Transient right limb muscle weakness was noted, with gradual recovery after rehabilitation treatment. Pathological examination confirmed the diagnosis of MOGHE (Figure 8).

**Figure 9:**
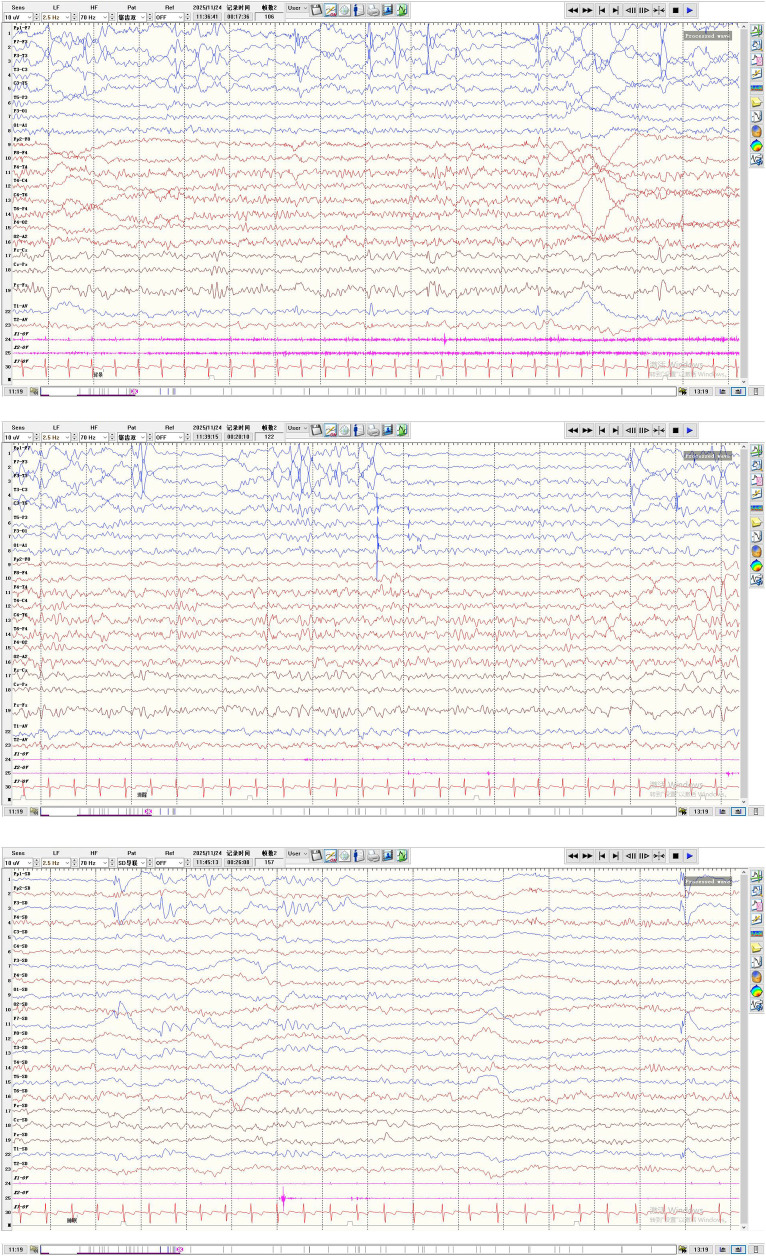
Postoperative follow-up VEEG (3 months after surgery): left frontal dominant slow wave and sharp wave discharges with contralateral occasional spread.

At 3 months postoperative follow-up, VEEG showed abnormal discharges limited to the left frontal lobe, with only occasional scattered abnormalities in other brain regions (Figure 9). The child's intellectual and language abilities were significantly improved compared with preoperative status, with the ability to babble and sing children's songs; no clinical seizures were observed by the parents. At 1 year postoperatively, vigabatrin was discontinued, with only oral sodium valproate continued; no epileptic seizures occurred, and neurodevelopment progressed continuously.

This study was approved by the Ethics Committee of Hebei Province of Children's Hospital, and the child's parents signed informed consent for the use of case data in academic publication.

## Discussion

MOGHE is a newly established neuropathological entity identified in surgical specimens from patients with drug-resistant epilepsy (7), officially included in the ILAE focal cortical dysplasia (FCD) classification system in 2022 (9). It has unique clinical, imaging, electrophysiological, and pathological characteristics, and treatment decision-making for infantile-onset MOGHE-associated epilepsy faces great challenges, with surgical timing selection as a core difficulty. Based on this case of early surgical intervention and a review of the relevant literature, this paper discusses surgical timing for MOGHE-associated childhood epilepsy, particularly infantile-onset cases.

### Clinical and pathological characteristics of MOGHE: why do they pose treatment challenges?

MOGHE-associated epilepsy predominantly onsets in infancy, most commonly involving the frontal lobe, and often presents as drug-resistant epileptic spasms with symptomatology lacking clear lateralizing value (10). VEEG manifestations are complex, typically showing generalized or multifocal abnormal discharges including bilateral spike-and-wave complexes, hypsarrhythmia-like changes, and characteristic “delta brush” or low-voltage fast activity in perilesional areas (13). This electrophysiological “generalization” or “multifocality” contradicts traditional epileptogenic zone localization concepts, increasing preoperative evaluation difficulty and hindering accurate delineation of the epileptogenic zone.

The contradictory phenomenon of “superficial seizure control” (e.g., resolution of clinical seizures after vigabatrin administration in this case) and “persistent brain injury” (continuous neurodevelopmental regression) is a key clinical feature of MOGHE, suggesting persistent subclinical epileptic activity or underlying epileptic encephalopathy (11).

Conventional cranial MRI is limited in detecting MOGHE; positive findings may only include mild cortical thickening, blurring of the gray-white matter junction, or T2/FLAIR hyperintensity at the corticomedullary junction (11, 14, 15), with some cases showing completely normal MRI. Fused PET-MRI may also lack typical epileptogenic zone hypometabolism, or only show mild, atypical metabolic changes. This “occult” or “atypical” imaging feature renders traditional surgical decision-making models (reliant on definite structural abnormalities) ineffective (9, 14, 15).

Pathological diagnosis is the gold standard for MOGHE, characterized by diffuse oligodendroglial hyperplasia in the white matter and deep cortical layers (Olig2-positive cell density >2,200/mm^2^), accompanied by gray-white matter junction blurring and white matter neuronal heterotopia. These changes may be associated with somatic SLC35A2 gene mutations (8, 16, 17). Abnormal oligodendroglial hyperplasia may create a highly epileptogenic microenvironment by interfering with normal myelination and neuron-glial cell interactions, partially explaining the extensive electrophysiological abnormalities and incomplete pharmacotherapeutic response.

### Limitations and reflections of traditional surgical timing standards in the treatment of infants with MOGHE

The current general surgical timing standard for drug-resistant epilepsy requires inadequate seizure control despite adequate and standardized treatment with two appropriate antiepileptic drugs for at least 1 year (12). This standard aims to balance surgical benefits and risks and avoid unnecessary surgery. However, strict adherence to this “waiting period” for MOGHE-associated infantile epilepsy may lead to irreversible neurological damage.

Although recurrent seizures have traditionally been linked to neuronal death and structural brain injury (18), emerging evidence suggests more complex mechanisms underlying epilepsy-related cognitive impairment. Recent studies indicate that seizure activity disrupts hippocampal long-term potentiation and impairs gamma oscillations—processes essential for learning, memory, and synaptic plasticity during critical developmental windows (19, 20). In early-onset childhood epilepsy, such functional disruptions may interfere with neural circuit maturation before overt structural damage occurs, contributing to progressive cognitive decline and neurodevelopmental delay (21, 22). Thus, a prolonged preoperative waiting period based solely on pharmacotherapeutic response may fail to prevent these activity-dependent developmental disturbances, and early surgical intervention should be considered to mitigate potential long-term cognitive consequences and optimize neurodevelopmental outcomes.

Infancy is a critical window for brain plasticity; MOGHE-associated epileptic encephalopathy continuously impairs the developmental foundation of cognitive, language, and social functions. Studies focusing on infants have shown that persistent epileptic activity—even infrequent clinical or subclinical seizures—can severely disrupt normal neural network development and plasticity during critical brain maturation windows (23–25). Honda and Otsuki (23) reported that among 12 infants with hemimegalencephaly who underwent hemispherotomy at a mean age of 4.3 months, postoperative seizure freedom was associated with significantly higher developmental quotients, and shorter preoperative seizure duration correlated with better developmental outcomes.

Subsequent large-scale studies by Iwasaki et al. (25) confirmed that postoperative seizure freedom was a key determinant of developmental progress in 75 children who underwent epilepsy surgery under 3 years of age. Makridis et al. (3) found that 84.6% of infants operated on within the first six months of life had pre-existing cognitive impairment, underscoring the urgency of early intervention. The latest meta-analysis by Hendrawan et al. (5) also emphasized that postoperative seizure freedom is crucial for infant developmental outcomes. This case demonstrates that significant intellectual and language regression can occur despite “adequate” seizure control in MOGHE-associated epilepsy.

Some children with MOGHE may show initial good responses to specific drugs (e.g., vigabatrin for infantile spasms), but “reduced seizures” do not equate to “encephalopathy control”. Concurrently, neurodevelopmental stagnation or regression indicates ongoing epileptic encephalopathy. The time spent waiting for “adequate” pharmacotherapy may coincide with the period of most rapid neurological function loss; thus, continuous neurodevelopmental regression is a more important intervention indicator than visible clinical seizures.

MOGHE is a diffuse developmental abnormality primarily involving white matter lesions, with an epileptogenic network often more extensive than suggested by imaging. Literature shows a high seizure recurrence rate after limited lesion resection, while more thorough anatomical disconnection surgery (e.g., frontal lobectomy) achieves higher seizure-free rates (10). This indicates a paradigm shift in treatment goals from resecting a single “epileptogenic point” to disconnecting the entire “epileptogenic network”. Early surgery aims to maximize interruption of abnormal discharge spread and create opportunities for normal brain development.

### Theoretical basis and decision-making considerations for early surgical intervention

Based on the above characteristics of MOGHE, surgical timing decision-making for infants should shift from the traditional “post-drug failure” model to an active “developmental protection” intervention strategy, based on comprehensive multimodal evaluation. The core indication for active preoperative evaluation is definite neurodevelopmental regression—the strongest signal to initiate surgical assessment. Progressive intellectual, language, or motor regression identified via developmental scale assessment should be regarded as an equally important intervention indicator as frequent clinical seizures.

Although VEEG may show extensive manifestations, careful analysis of long-term VEEG can often identify background activity asymmetry, slow wave foci, or dominant interictal discharge regions, providing key evidence for surgical lateralization; intracranial VEEG may further accurately delineate the seizure onset zone. Finally, imaging evaluation should adopt high-resolution MRI with advanced post-processing techniques and actively utilize special sequences (e.g., T1-CHESS) to detect subtle structural abnormalities (15); PET-MRI fusion should focus on analyzing the correlation between metabolic changes and electrophysiologically abnormal areas.

Surgical decision-making must also fully consider the unresolved etiological conundrum in MOGHE: whether neurodevelopmental impairment stems from intrinsic structural and molecular abnormalities of MOGHE itself (a developmental encephalopathy) or persistent epileptic activity (an epileptic encephalopathy). This distinction fundamentally defines surgery as a potentially curative intervention (for seizure-driven damage) or a palliative measure (for primary developmental deficits) (26). Thus, definite, progressive neurodevelopmental regression remains a core indication for active preoperative evaluation but should not be the sole factor given this etiological uncertainty—progressive regression alone is clinically insufficient rationale for epilepsy surgery.

Building on the meta-analysis by Alim-Marvasti et al. (27), additional critical factors must be integrated into the decision-making framework: (1) persistent electrophysiological abnormalities (even subclinical epileptiform discharges or focal/dominant hemisphere dysrhythmia) on long-term VEEG despite adequate pharmacotherapy; (2) multimodal imaging evidence of a focal or laterally restricted epileptogenic network (e.g., subtle structural abnormalities on high-resolution MRI/T1-CHESS or metabolic alterations on PET-MRI fusion correlated with electrophysiological deficits); (3) exclusion of reversible genetic or metabolic etiologies for developmental regression via comprehensive genetic and metabolic testing.

Although VEEG may show extensive or generalized manifestations, careful analysis of long-term scalp or intracranial VEEG can often identify background activity asymmetry, slow wave foci, or dominant interictal discharge areas, providing key lateralization evidence for the epileptogenic zone—an essential factor to confirm that surgical disconnection/resection can target the primary source of epileptic activity. Imaging should adopt high-resolution MRI with advanced post-processing techniques and actively implement special sequences (e.g., T1-CHESS) to detect subtle structural abnormalities (15); PET-MRI fusion should focus on analyzing the spatial correlation between focal metabolic changes and electrophysiologically abnormal areas to further validate the link between epileptic activity and observed developmental decline.

In 2023, Liu et al. (11) conducted a retrospective analysis of 37 children with MOGHE-associated epilepsy combined with a literature review, showing that most cases onset in infancy and early childhood (especially before 3 years of age), with epileptic spasms as the most common seizure type, moderate to severe developmental delay in most patients, and favorable surgical prognosis (76.2% seizure-free postoperatively). This study suggests that MOGHE should be suspected in children with early-onset epilepsy, and active integration of imaging and electrophysiological evaluations combined with more thorough surgical strategies are necessary to improve long-term prognosis.

The final surgical decision should be made via multidisciplinary collaboration including neurologists, epileptic surgeons, neuroelectrophysiologists, neuroradiologists, and neuropathologists. The focus of evaluation includes: (1) whether the epileptogenic network is mainly limited to a unilateral, operable cerebral lobe (especially the frontal lobe); (2) whether the neurodevelopmental trajectory can still be improved with pharmacotherapeutic intervention; (3) the risk-benefit ratio between surgical functional risks and the developmental damage risks of non-surgical management. 

### Recommendations

Early surgical intervention should be actively considered when a child presents with the following conditions: (1) drug-refractory epileptic spasms or frequent focal seizures; (2) definite and progressive neurodevelopmental regression; (3) multimodal evaluation suggesting the epileptogenic network is mainly limited to the unilateral frontal lobe or other operable cerebral lobes; (4) failure of adequate pharmacotherapy (including potentially effective drugs such as vigabatrin) to reverse developmental regression.

Given the diffuse nature of MOGHE lesions, anatomical lobar disconnection with a more thorough surgical scope should be preferred to achieve better seizure control and developmental improvement, maximizing disconnection of the abnormal discharge network (10, 11). The ultimate goal of early surgery is not only seizure freedom but also removing obstacles to stagnant childhood neurodevelopment.

In the future, more prospective studies are needed to evaluate the exact impact of early surgery on long-term neurodevelopmental outcomes in children with MOGHE. Simultaneously, targeted therapies for SLC35A2 mutations (e.g., galactose supplementation) as postoperative adjuvant therapy or potential treatment for non-surgical patients warrant further exploration (8, 16).

In this case, the child underwent frontal lobectomy due to significant developmental regression only 3 months after seizure onset, prior to meeting traditional drug-resistant epilepsy criteria. Postoperatively, seizure freedom was achieved with significant neurological function improvement, providing strong clinical evidence for early surgical intervention in such patients. Larger sample studies with longer follow-up periods are needed to further verify the impact of early surgery on long-term neurodevelopmental outcomes in children with MOGHE.

## Conclusion

In summary, for MOGHE-associated drug-resistant epilepsy in childhood, surgical timing selection should abandon rigid “time window” criteria and adopt an individualized decision-making approach oriented by “developmental prognosis”. Early surgical intervention based on multimodal evaluation may effectively prevent irreversible neurodevelopmental damage and improve long-term clinical outcomes for children with MOGHE-associated epilepsy and progressive neurodevelopmental regression.

## Data Availability

The authors acknowledge that the data presented in this study must be deposited and made publicly available in an acceptable repository, prior to publication. Frontiers cannot accept a manuscript that does not adhere to our open data policies.
